# A malaria vaccine protects *Aotus* monkeys against virulent *Plasmodium falciparum* infection

**DOI:** 10.1038/s41541-017-0015-7

**Published:** 2017-05-22

**Authors:** Prakash Srinivasan, G. Christian Baldeviano, Kazutoyo Miura, Ababacar Diouf, Julio A. Ventocilla, Karina P. Leiva, Luis Lugo-Roman, Carmen Lucas, Sachy Orr-Gonzalez, Daming Zhu, Eileen Villasante, Lorraine Soisson, David L. Narum, Susan K. Pierce, Carole A. Long, Carter Diggs, Patrick E. Duffy, Andres G. Lescano, Louis H. Miller

**Affiliations:** 1 Laboratory of Malaria and Vector Research, National Institute of Allergy and Infectious Diseases, National Institutes of Health, Rockville, MD USA; 2 US Naval Medical Research Unit No. 6 (NAMRU-6), Callao, Peru; 3 Laboratory of Malaria Immunology and Vaccinology, National Institute of Allergy and Infectious Diseases, National Institutes of Health, Rockville, MD USA; 4 US Military Malaria Vaccine Program, Naval Medical Research Center, Silver Spring, MD USA; 5 Malaria Vaccine Development Program, U.S. Agency for International Development, Washington, DC USA; 6 Laboratory of Immunogenetics, National Institute of Allergy and Infectious Diseases, National Institutes of Health, Rockville, MD USA; 7 Present Address: Johns Hopkins Malaria Research Institute, Dept. Molecular Microbiology and Immunology, Johns Hopkins Bloomberg School of Public Health, Baltimore, MD USA

## Abstract

The *Plasmodium falciparum* protein, apical membrane antigen 1 forms a complex with another parasite protein, rhoptry neck protein 2, to initiate junction formation with the erythrocyte and is essential for merozoite invasion during the blood stage of infection. Consequently, apical membrane antigen 1 has been a target of vaccine development but vaccination with apical membrane antigen 1 alone in controlled human malaria infections failed to protect and showed limited efficacy in field trials. Here we show that vaccination with AMA1–RON2L complex in Freund’s adjuvant protects *Aotus* monkeys against a virulent *Plasmodium falciparum* infection. Vaccination with AMA1 alone gave only partial protection, delaying infection in one of eight animals. However, the AMA1–RON2L complex vaccine completely protected four of eight monkeys and substantially delayed infection (>25 days) in three of the other four animals. Interestingly, antibodies from monkeys vaccinated with the AMA1–RON2L complex had significantly higher neutralizing activity than antibodies from monkeys vaccinated with AMA1 alone. Importantly, we show that antibodies from animals vaccinated with the complex have significantly higher neutralization activity against non-vaccine type parasites. We suggest that vaccination with the AMA1–RON2L complex induces functional antibodies that better recognize AMA1 as it appears complexed with RON2 during merozoite invasion. These data justify progression of this next generation AMA1 vaccine towards human trials.

## Malaria: Inhibiting parasite invasion of red blood cells

A vaccine targeting a protein complex that allows malaria-causing parasite to enter red blood cells has been produced. Malaria caused by the parasite *Plasmodium falciparum* is an oft-deadly infectious disease without an effective vaccine. A team of researchers at the National Institutes of Health led by Prakash Srinivasan, currently at the Johns Hopkins Malaria Research Institute, United States, demonstrated the efficacy of a vaccine candidate that works by priming a host’s immune system to a parasitic protein complex required to form a junction with red blood cells, allowing entry and proliferation of the pathogen. The group’s vaccine conferred more effective protection in monkeys than prior candidates that targeted only one component of the parasitic protein complex. This research warrants a closer look into how this candidate, and others targeting the protein complex, can be used to prevent malaria in humans.

## Introduction

Malaria caused by *Plasmodium falciparum* (*Pf*) remains one of the most deadly infectious diseases in the world. The disease afflicts young children and pregnant women disproportionately. The recent development of resistance to front-line antimalarial drugs underscores the urgent need to develop an effective vaccine. Clinical disease is caused by the asexual forms of the parasite that replicate within red blood cells (RBCs). Therefore, a vaccine that blocks the parasites from entering the RBC could prevent disease. People living in malaria endemic countries develop resistance to clinical disease after years of repeated exposure to the parasites. A recent study in Mali found no difference in time-to-infection in both children and adults, indicating no pre-erythrocytic immunity, but adults were significantly protected from clinical disease.^1^ Interestingly, IgG purified from malaria-exposed adults are able to suppress growth of parasites when transferred to non-immune individuals,^2, 3^ suggesting that antibodies play an important role in conferring clinical immunity. These observations indicate the possibility of developing a vaccine that would accelerate the acquisition of protective immunity to disease in children. Such a vaccine will have an enormous impact on reducing mortality and disease severity in children and pregnant women.

The high antibody titers against AMA1 in malaria-exposed individuals, its surface expression and ability of anti-AMA1 antibodies to block invasion in vitro led to AMA1 being a leading vaccine candidate.^4–8^ The high level of polymorphisms in AMA1 was thought to present a major challenge to the development of AMA1 as an effective vaccine. However, recent studies have demonstrated that a small number of alleles (as few as four natural alleles or three synthetic alleles covering polymorphisms), could be sufficient to cover all major polymorphisms.^9–12^ Nevertheless, the inability of an AMA1 subunit vaccine to protect against vaccine-type parasites in controlled infection studies and poor efficacy in a field trial despite inducing high titer antibodies have dampened enthusiasm for an AMA1 vaccine.^13–16^ It is possible that these subunit vaccines are unable to induce a threshold concentration of functional, protective antibodies and therefore improving the quality rather than just the quantity or breadth of antibodies may improve vaccine efficacy.

Our approach to improving the quality of antibodies elicited by AMA1 vaccination is to develop a vaccine that more closely mimics the AMA1 structure on the invading merozoite. *Plasmodium* spp. merozoites utilize a sophisticated mechanism for invasion of RBCs by secreting their own receptor (the RON complex) on to the plasma membrane of the target RBC.^17, 18^ A 49-amino acid conserved region of Rhoptry neck protein 2 (RON2) on the RBC membrane binds to merozoite surface apical membrane antigen 1 (AMA1), a step that commits the parasite for invasion.^17–20^

Assuming that the immune system must recognize the AMA1-RON2 complex to effectively block invasion in vivo, we developed and recently demonstrated that vaccination with PfAMA1–RON2L complex in rats induced qualitatively better invasion inhibitory antibodies against *Pf* as compared to the antibodies elicited by vaccination with AMA1 alone.^21^ Importantly, vaccination with a *P. yoelii* (*Py*) AMA1–RON2L complex but not AMA1 alone provided complete antibody-dependent protection against lethal *Py* challenge in mice,^21^ suggesting that the antibody response was shifted towards functionally important epitopes.

Here we determined whether vaccination with the PfAMA1–RON2L complex could better protect non-human primates against virulent *P. falciparum* FVO strain malaria as compared to vaccination with AMA1 alone. This non-human primate model of human malaria has been used to assess the protective efficacy of malaria vaccine candidates including AMA1, which by itself shows moderate efficacy.^6^ In this study, we found that four of eight animals immunized with the AMA1–RON2L complex were parasite-free until end of study on day 40 after challenge with infected RBCs. An additional three of eight animals had a substantial delay (>25 days) in onset of parasitemia. In contrast, none of the eight animals immunized with AMA1 alone were protected from infection and only one animal had a delay in patency. Importantly, the improved efficacy of the AMA1–RON2L complex vaccine over AMA1 alone was not due to a quantitative change in the overall antibody levels but rather a qualitative shift in the proportion of AMA1-specific antibodies that block invasion. Interestingly, the complex also enhanced the immunogenicity of certain conserved epitopes as observed by a significant increase in the neutralization of heterologous 3D7 and GB4 parasites. Our data suggest that it is possible to induce sufficient levels of neutralizing antibodies to confer protection and that a vaccine containing a limited number of AMA1 alleles in complex with the conserved RON2L peptide may protect against all parasites.

## Results

### Evaluation of vaccine efficacy of AMA1 alone vs. AMA1–RON2L complex

The goal of this study was to test the hypothesis that vaccination with AMA1–RON2L complex provides superior protection than AMA1 alone against a virulent *P. falciparum* challenge. Recombinant AMA1 corresponding to the FVO strain and a conserved RON2L peptide were used in this study. Recombinant AMA1 appeared to be folded correctly as demonstrated by reactivity of a mAb to a conformational epitope in AMA1 (Supplementary Fig. S1a). RON2L binding to AMA1 was confirmed by surface plasmon resonance (SPR; Supplementary Fig. S1b).

**Fig. 1:**
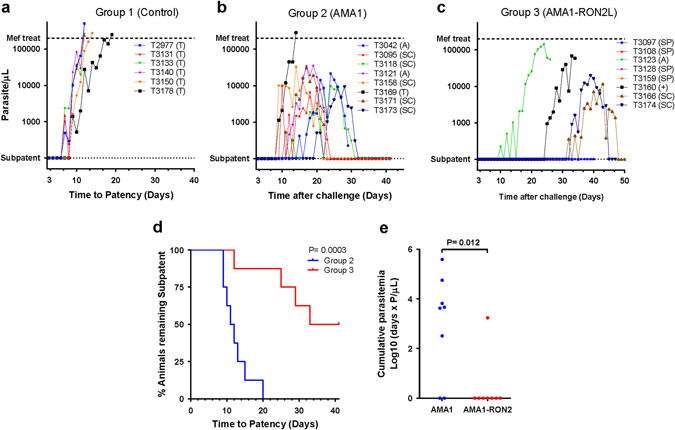
PfAMA1–RON2L complex protects against virulent *P. falciparum* challenge. **a**–**c** Time course of parasitemia after challenge with 10^4^ *Pf* FVO strain infected RBCs for animals in the control, adjuvant only (**a**) Group 1), AMA1 (**b**) Group 2) and AMA1–RON2L complex (**c**) Group 3). The *dashed line* at the *top* indicates limit of parasitemia (200,000 parasites per μL) at which time animals were treated with the antimalarial drug, mefloquine. The *dotted line* at the bottom indicates the absence of thin smear detectable parasites. T, treatment due to high parasitemia; A, treatment due to anemia; +, a single animal found dead possibly due to anemia; SC, self-cured; SP: animals that remained thin-smear negative until day 41 after challenge when the study was terminated. (**d**) Kaplan–Meier plot of time-to-patency of animals in Groups 2 and 3. Log-rank was performed to compare time-to-patency (parasite positive by thin blood smear) of animals between Group 2 and Group 3 by the Mantel-Cox test (*P* = 0.0003). (**e**) The cumulative parasitemia up to day 14, the day on which the first animal in Group 2 was treated, between Groups 2 and Group 3 by the Mann–Whitney test (*P* = 0.012)

*Aotus nancymaae* monkeys were randomized into three groups corresponding to adjuvant control (Group 1, *n* = 6), AMA1 alone (Group 2, *n* = 8) and AMA1–RON2L complex (Group 3, *n* = 8). Each group received three doses of the corresponding antigen formulated with complete or incomplete Freund’s adjuvant. To evaluate protective efficacy, freshly collected *Pf* FVO strain parasites (10^4^ infected RBCs) obtained from a donor monkey were administered intravenously 4 weeks after the final vaccination. Randomization of animals was maintained throughout the period of parasite challenge ensuring consistency in parasite viability between the three groups. Parasitemia was measured everyday on thin smears (Fig. 1a–c) and hematocrit was followed every other day (Supplementary Fig. S2a). From previous studies, *Pf* FVO infection of *Aotus* monkeys is known to be highly virulent.^6^ Consistently, all animals in Group 1 (adjuvant control) became slide-positive by day nine post-challenge, and all required treatment for high parasitemia (Fig. 1a).

The protocol-specified primary analysis showed significantly higher efficacy (as measured by time-to-patency) of AMA1–RON2L complex vaccine (Group 3) compared to animals vaccinated with AMA1 alone (Group 2) (*P* = 0.0003, Log-rank (Mantel-Cox) test, Fig. 1d). While all animals (eight of eight) in Group 2 became infected, 50% of the animals (four of eight) in Group 3 remained thin smear negative until end of study (day 40 post-challenge, Fig. 1b, c). Furthermore, the time-to-patency in three of the remaining four animals in Group 3 was substantially longer (>25 days) than all animals in Group 2 (Fig. 1d). Polymerase chain reaction (PCR) was performed on blood samples collected from the four Group 3 animals that remained thin smear-negative at the end of the study to detect low-level parasitemias. All were PCR-negative, indicating that these animals cleared the initial intra venous challenge and were sterilely protected (Supplementary Fig. S2b). Secondary analysis comparing log_10_ cumulative parasitemia up to day 14, the first day of treatment for any animal in Groups 2 and 3, also showed a significantly lower parasite load in animals vaccinated with the AMA1–RON2L complex (*P* = 0.012, Mann–Whitney test) (Fig. 1e). Of the animals that became infected, parasitemia in one of the animals in Group 2 reached 200,000 parasites per μL, a level that required treatment. Two animals in Group 2 and two in Group 3 were treated for anemia (Fig. 1b, c), a complication that occurs in this *Aotus* model of *Pf*.^6^ Given that in human trials of malaria vaccines (e.g., Phase 1/2 efficacy study), antimalarial treatment is generally administered as soon as parasites are detected in the blood film, an effective vaccine has to induce sterile protection or a significant delay in time-to-patency. By this criterion, given that four animals were subpatent (SP) and an additional three of four animals showed a significant delay in time-to-patency as compared to animals vaccinated with AMA1 alone, AMA1–RON2L complex appears to be a highly effective vaccine. This high level of efficacy induced by AMA1–RON2L complex vaccine is similar to the efficacy observed in a recent *P. falciparum* RH5 vaccine study using the same adjuvant.^22^

### Correlation of vaccine-induced antibody responses with protection

**Fig. 2:**
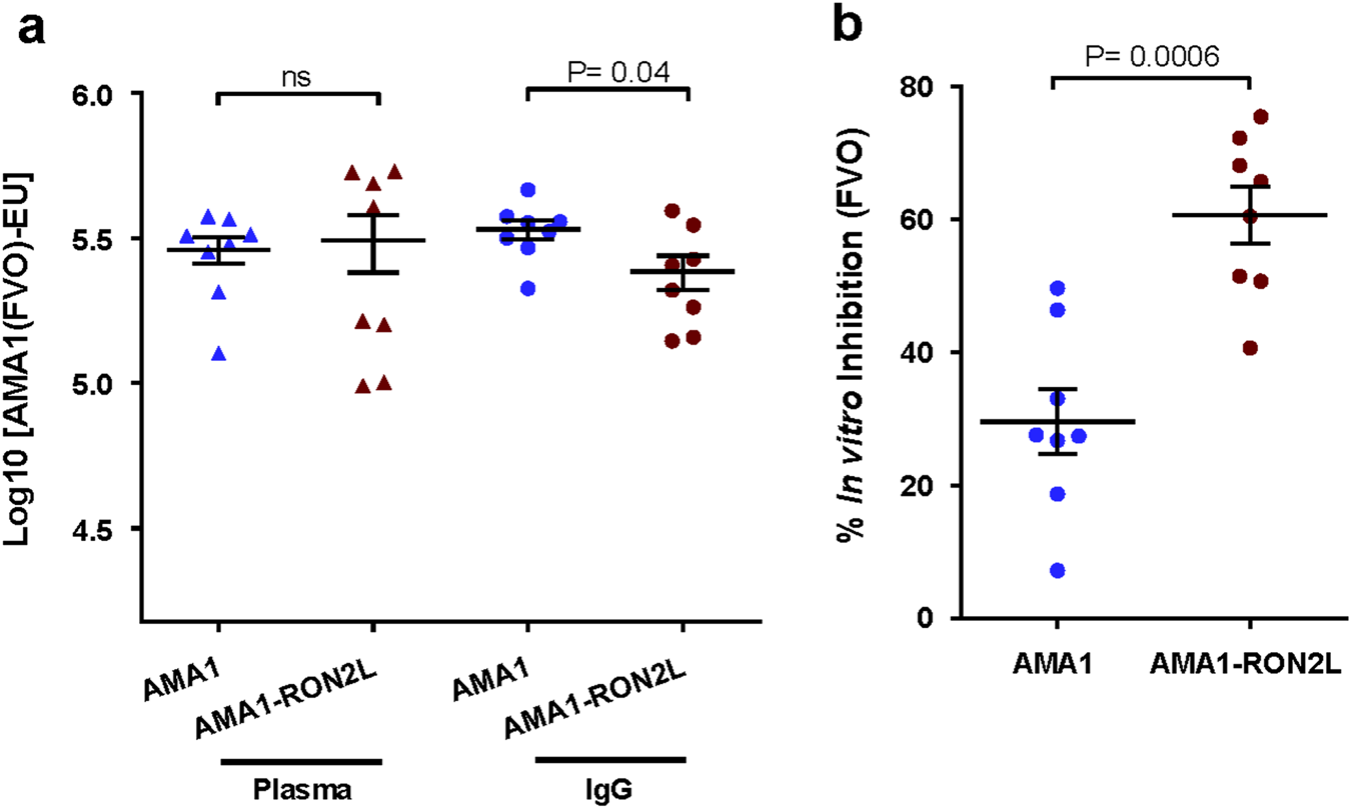
Vaccination with AMA1–RON2L complex induces a shift in the quality of blocking antibody. **a** FVO AMA1-specific antibody levels in plasma and purified IgG. ELISA was performed on individual samples collected before the day of challenge. Arbitrary ELISA units based on standard curves were generated to compare anti-FVO AMA1 antibody levels in plasma and purified IgG of Group 2 and Group 3 animals by the Mann–Whitney test (plasma: *P* = 0.854; IgG: *P* = 0.045). Data are shown for individual animals and represented as mean ± SEM. (**b**) In vitro GIA using purified IgG against the homologous FVO strain. Total IgG from each animal was tested at 2.5 mg mL^−1^ final concentration and inhibitory activity was compared between Group 2 and 3 by Mann–Whitney test (*P* = 0.0006). Data shown are from two independent experiments represented as mean ± SEM. Antibody data are from plasma samples collected 4 weeks after last vaccination (before parasite challenge)

First we measured total IgG levels in the plasma and found that they were similar between Group 2 and Group 3 animals (Supplementary Fig. S2c). Next, we examined AMA1-specific antibody levels in plasma and found that the levels of AMA1-specific antibody were similar between Group 2 and 3 animals (Fig. 2a). Our earlier results using a virulent *Py* mouse malaria model showed that it is not the quantity, but rather the quality of AMA1-specific antibodies that determines protection.^21^ Based on these findings we hypothesized that the significant differences in vaccine efficacy between Group 2 and 3 animals maybe due to a difference in the quality of anti-AMA1 antibodies induced by the two vaccines. To test this hypothesis, we assessed the functional activity of the antibodies using the well-established one-cycle, in vitro growth inhibition assay (GIA), which essentially measures levels of merozoite neutralizing activity.^23^ Consistent with our hypothesis, IgG purified from Group 3 animals showed significantly higher neutralization of challenge-strain FVO parasites as compared to IgG from Group 2 at 2.5 mg mL^−1^ total IgG (Fig. 2b). This result despite the levels of AMA1-specific antibodies in the purified IgG (all adjusted at 10 mg/mL regardless of total IgG concentrations in the original plasma) being slightly lower in the animals vaccinated with AMA1–RON2L as compared to that of animals immunized with AMA1 alone (Fig. 2a), strongly suggests qualitative differences in the AMA1-specific antibodies between these two groups.

**Fig. 3:**
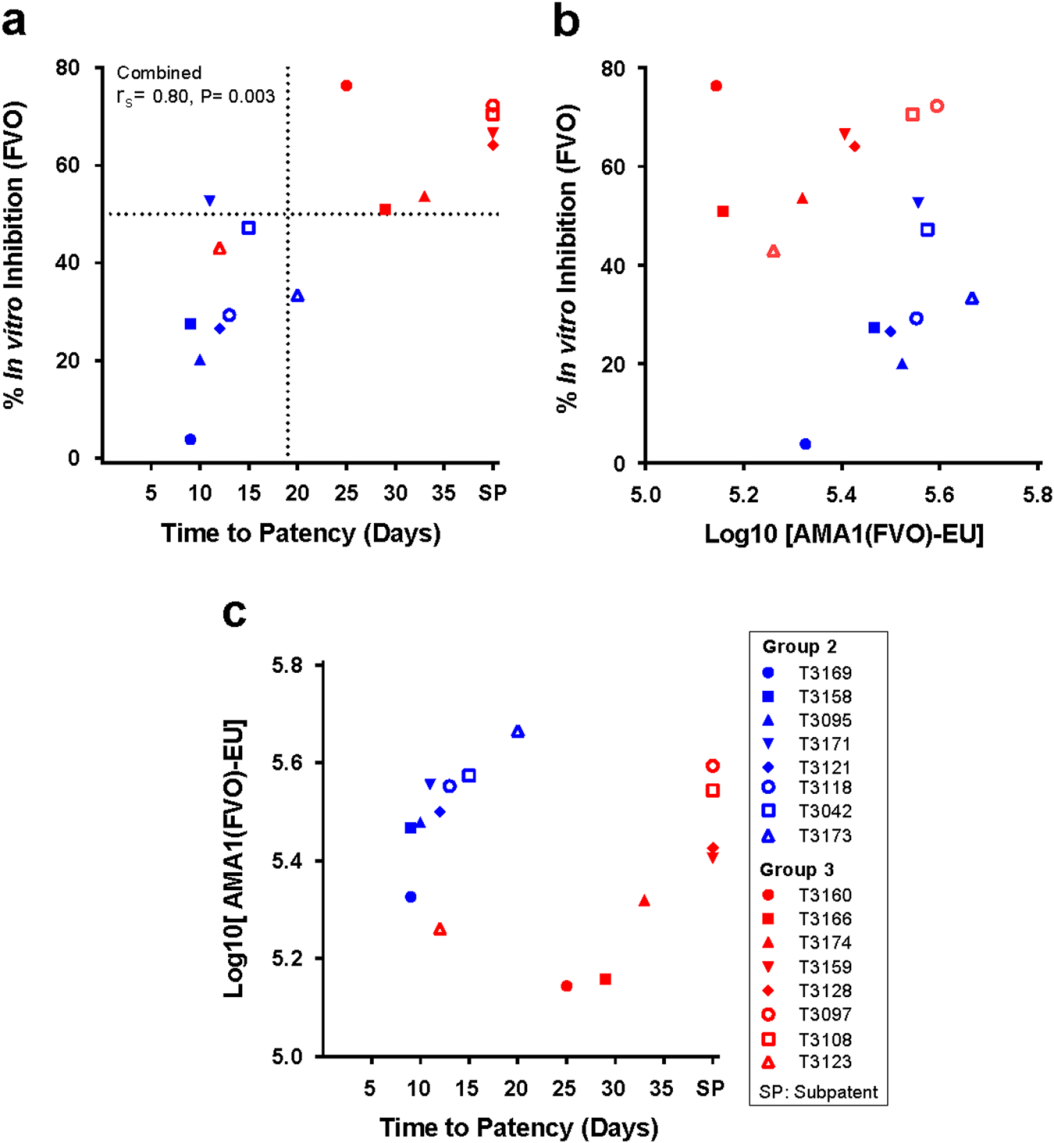
Complex-induced enhancement in antibody quality is associated with protection. **a** The relationship between time-to-patency and in vitro growth inhibitory activity of purified IgG for the 16 animals in Groups 2 (*blue*) and 3 (*red*). Spearman’s rank correlation coefficient (*r*_s_) and *P* value are shown for the relationship of time-to-patency and in vitro growth inhibitory activity of purified IgG from the same animals. The *horizontal dotted line* represents the 50% GIA and the *vertical dotted line* separates the animals that either had a significant delay in patency (>15 days) or were SP until the end of the study. **b** Anti-PfAMA1(FVO) antibody levels do not correlate with in vitro growth inhibitory activity (*r*_s_ = −0.082, *P* = 0.76). **c** The relationship between anti-PfAMA1(FVO) antibody levels and time-to-patency shows no correlation (*r*_s_ = −0.008, *P* = 0.94). GIAs were performed at 2.5 mg mL^−1^ total IgG from each immunized animal

A significant association between GIA at 2.5 mg mL^−1^ total IgG and time-to-patency, the primary analysis for correlate of protection, was observed across both Groups 2 and 3 (Fig. 3a). However, there was no correlation between vaccine-induced AMA1 antibody titer and neutralization in vitro or time-to-patency in vivo in the two groups combined, but correlates are apparent for each group (Fig. 3b, c). Importantly, after adjusting for anti-AMA1 antibody levels, IgG from Group 3 animals showed significantly higher inhibition than Group 2 (*P* < 0.001 by a multiple regression analysis) (Fig. 3b), consistent with our hypothesis that vaccination with the AMA1–RON2L complex induced a qualitative shift in the antibody response. Interestingly, seven of eight animals that were either sterilely protected or had *a* > 25-day delay in time-to-patency also had GIAs > 50% (Fig. 3a). We also noticed that despite similar GIA at 2.5 mg mL^−1^ total IgG, animals T3042, T3166, T3171 and T3174 had very different in vivo outcomes (time-to-patency) (Fig. 3a). While our data show a strong correlation between in vivo protection and GIA, caution must therefore be exercised before extrapolating in vitro GIA to predict in vivo outcomes.

### Levels of AMA1–RON2L blocking antibodies correlate with protection

**Fig. 4:**
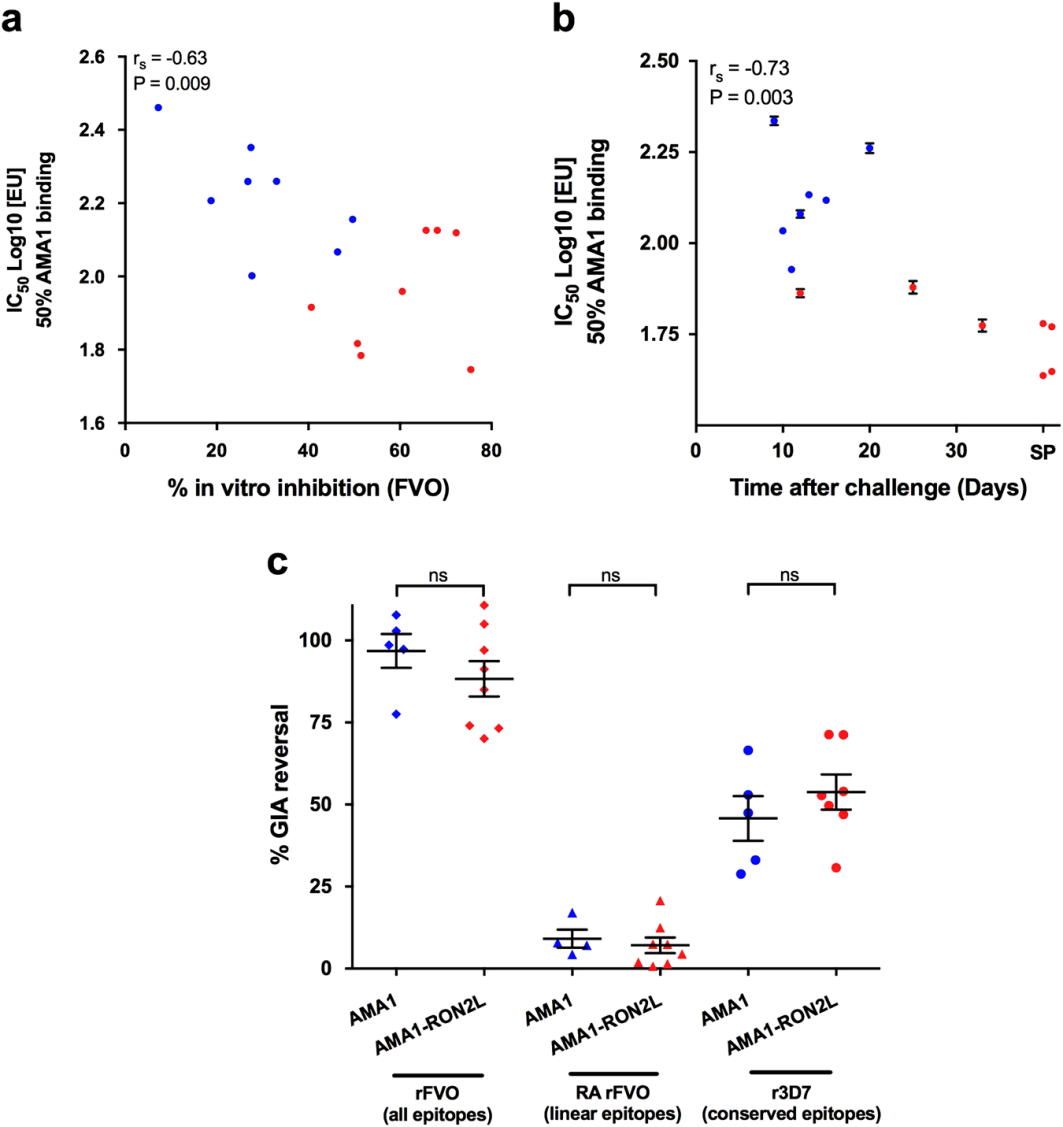
Levels of AMA1-RON2 blocking antibodies correlate with protection. **a** The relationship between levels of AMA1-RON2 blocking antibodies (IC_50_ Log10[EU]) and in vitro neutralization activity of the corresponding purified IgG. **b** The relationship between levels of AMA1-RON2 blocking antibodies (IC_50_ Log10[EU]) in the plasma and time to infection after challenge in these animals. Spearman’s rank correlation coefficient (*r*_s_) and *P* value are shown for each comparison. **c** Growth inhibitory antibodies largely target conformational epitopes in AMA1. The conformational and allele-specific dependency of antibodies to block invasion was assessed by measuring the ability of recombinant AMA1 to block the inhibitory activity of IgG. Saturating concentrations (2 μM) of recombinant FVO AMA1 (rFVO), reduced and alkylated FVO AMA1 (RArFVO) or 3D7 AMA1 (r3D7) were pre-incubated with IgG before assessing their GIA activity against FVO strain parasites. The amount of IgG from each animal was chosen such that they had 40–50% GIA before recombinant proteins were added and four or more animals each from Groups 2 and 3 were tested. ns, not significant

Next we assessed the levels of antibodies that block the binding of AMA1 to RON2L. We have previously demonstrated that AMA1 mAbs binding near the hydrophobic pocket block the binding of RON2L and vaccination with the complex induce a higher level of such blocking antibodies.^18, 24^ A competition enzyme-linked immunosorbent assay (ELISA) was performed and IC_50_ was calculated to determine the levels of AMA1–RON2L blocking antibodies in both plasma and purified IgG (Supplementary Fig. S3a, b). Interestingly, the levels of blocking antibodies in the purified IgG were much higher in animals immunized with the AMA1–RON2L complex and strongly correlated with their invasion blocking activity (Fig. 4a). More importantly, levels of these blocking antibodies in the plasma of animals significantly correlated with protection (Fig. 4b), suggesting that the improved vaccine efficacy in Group 3 is, at least in part, due to the complex inducing antibodies that interfere with an important function of AMA1-RON2 during merozoite invasion. The enhancement in the activity of the antibodies however does not appear to be due to an increase in their avidity (Supplementary Fig. S3c).

### Neutralizing antibodies induced by the AMA1–RON2L complex do not discriminate AMA1 and AMA1–RON2L complex in vitro

We assessed if addition of saturating concentration of recombinant AMA1 proteins blocked the neutralizing activity of the antibody in the GIA using homologous FVO strain parasites. The addition of recombinant FVO AMA1 almost completely blocked the neutralizing activity of both Group 2 and 3 IgG (Fig. 4c). In contrast, reduced and alkylated FVO protein failed to block the GIA-neutralizing activity of the IgG from both groups (Fig. 4c). Taken together, these observations suggest that the specificity of antibodies induced by vaccination with AMA1–RON2L is primarily due to contacts of the antibodies with AMA1 not RON2L and that these contacts are sensitive to the conformation of AMA1. Recombinant heterologous 3D7 AMA1 was able to only partially block the GIA activity of the IgG from both AMA1 and AMA1–RON2L immunized animals (Fig. 4c), confirming earlier observations that polymorphisms in AMA1 are targets of functional antibodies. We also observed a slightly higher GIA reversal in the AMA1–RON2L complex group compared to AMA1 alone using heterologous r3D7 AMA1 along with a corresponding decrease in GIA reversal when using homologous rFVO AMA1. However these differences were not statistically significant (Fig. 4c).

### Vaccination with AMA1–RON2L complex induces significantly higher levels of broadly neutralizing antibodies

**Fig. 5:**
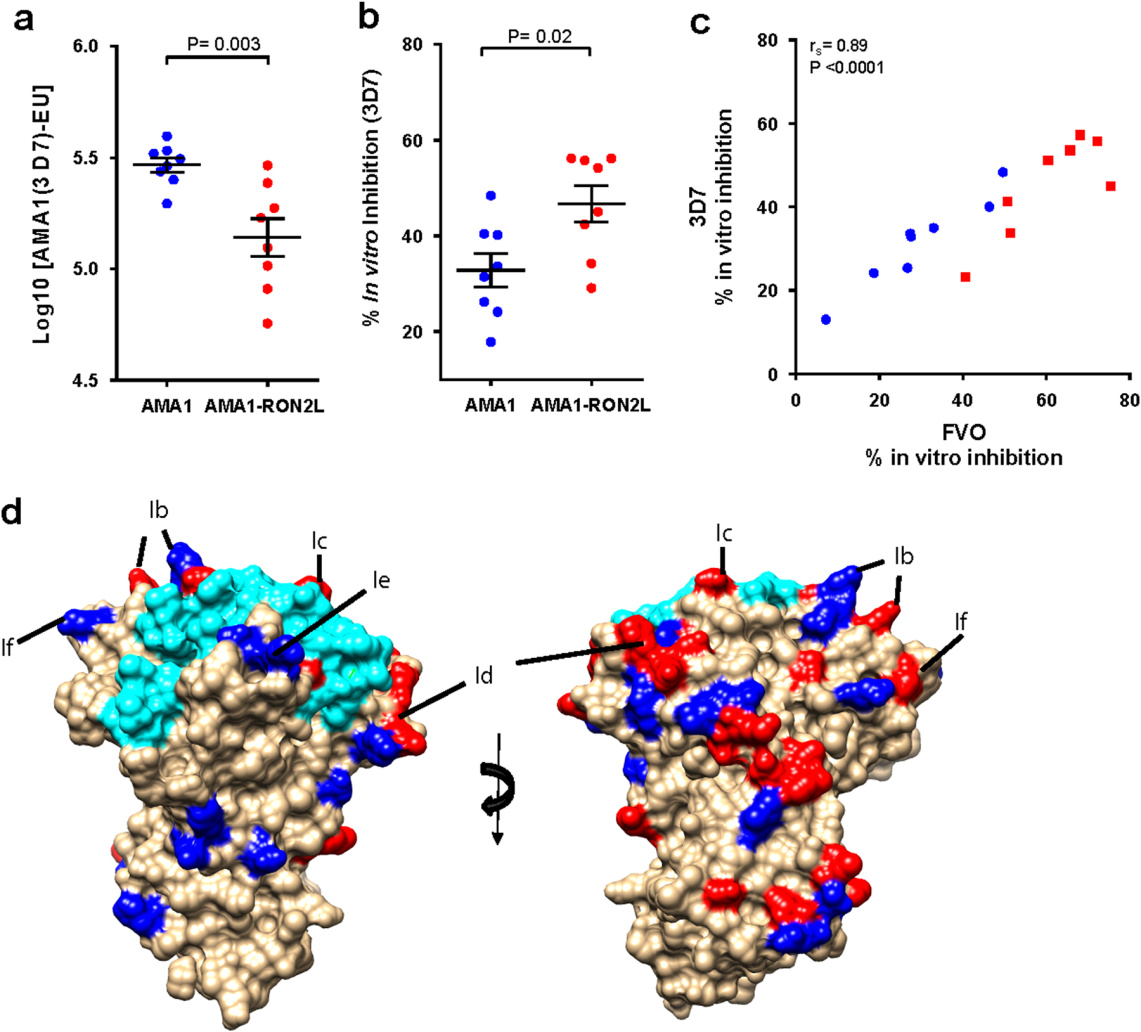
AMA1–RON2L complex induces an increase in the proportion of antibodies targeting conserved epitopes. **a** 3D7 AMA1-specific antibody levels in purified IgG were measured by ELISA. Arbitrary ELISA units based on standard curves were generated to compare anti-3D7 AMA1 antibody levels in purified IgG of Group 2 and Group 3 animals by Mann–Whitney test (*P* = 0.003). **b** In vitro GIA using purified IgG against the heterologous 3D7 strain. Total IgG from each animal were tested at 2.5 mg mL^−1^ final concentration and inhibitory activity was compared between Group 2 and 3 by the Mann–Whitney test (*P* = 0.02). **c** Correlation of growth inhibitory activity of IgG from Group 3 (*red squares*) and Group 2 (*blue circles*) between homologous FVO parasites and heterologous 3D7 parasites (*blue circles*) (*r*_s_ = 0.89, *P* < 0.0001). **d** Structural representation of AMA1 (*brown*) bound to RON2L (*cyan*) (PDB ID: 3ZWZ).^20^ The polymorphic residues that are conserved between FVO, 3D7 and GB4 parasites are shown in *dark blue* and residues that differ are shown in *red*. The *black lines* indicate the respective loops in AMA1 (Ib, Ic, Id, Ie and If) surrounding the RON2 binding site

The ability of recombinant heterologous 3D7AMA1 to partially block the GIA against FVO parasites prompted us to assess whether or not these antibodies can neutralize non-vaccine-type parasite strains. Previous studies have demonstrated strong strain-dependent neutralization by AMA1-specific antibodies.^25^ Surprisingly, at 2.5 mg mL^−1^ total IgG, 3D7 strain parasites were inhibited significantly higher by Group 3 than Group 2, despite overall lower levels of antibodies recognizing the 3D7 AMA1 (Fig. 5a, b). Similarly, neutralization of GB4 parasites, expressing another heterologous AMA1 allele, was higher for pooled total IgG at 2.5 mg mL^−1^ from Group 3 than Group 2 animals (Supplementary Fig. S4a, b). Taken together, these results suggest that the qualitative shift in the antibody response induced by the AMA1–RON2L complex also enhanced the responses to functionally important conserved epitopes in AMA1. A strong correlation in antibody levels and GIA between homologous FVO and heterologous 3D7 parasites for each animal is also observed (Fig. 5c). However, the percent inhibition in the GIA was lower for the 3D7 strain as compared to the FVO strain for each group (Fig. 5c). Interestingly, neutralization of heterologous 3D7 parasite by Group 3 IgG is higher than inhibition of homologous FVO parasite by Group 2 IgG (Figs. 2b and 5b). The lower levels of GIA against heterologous 3D7 compared to homologous FVO parasite is likely due to polymorphisms that are not conserved between these parasites (Fig. 5d).

## Discussion

The lack of efficacy of AMA1 vaccines in human trials has been attributed to insufficient amounts of antibody induced by the vaccines and the polymorphisms between vaccine-type and parasite strains in the field. However, no protection was seen in CHMI studies against a homologous parasite challenge either through infected mosquito bite^13^ or a direct blood-stage parasite challenge.^16, 26^ The lack of vaccine efficacy in these human clinical trials suggests that the failure to protect is not due to polymorphisms. Rather, may reflect the inability of the current AMA1 subunit vaccines to induce a threshold concentration of functional antibodies despite the overall high antibody titers. Our data suggest that vaccination with AMA1–RON2L complex enhances the proportion of the neutralizing antibodies without affecting the overall AMA1 antibody titers. Importantly, this qualitative shift in the immune response induced by the complex led to a substantial improvement in overall vaccine efficacy (87.5% in Group 3 vs. 25% in Group 2) in this non-human primate model of virulent *P. falciparum* malaria. This high level of protection has not been observed previously (e.g., PfAMA1 alone, PfMSP1, PfMSP3 and PfEBA175)^6, 7, 27^ in this animal model except with PfRH5 using the same adjuvant.^22^

Why did the parasitemia rise 26, 30 and 34 days after challenge? Between the inoculation and the time of rise in parasitemia, no parasites were observed on the thin blood films. One possibility for the rise in parasitemia from non-detectable levels to high levels was the appearance of mutations in AMA1, but that was not found in these breakthrough parasites (not shown). A second possibility is that the antibodies induced by the vaccine were successful in controlling the parasite load to levels below detection by blood smear. It is known that persistent, low-level infection causes no disease and the anti-AMA1 antibody levels drop during the period at the same rate as children who were not infected.^28^ This may have allowed the highly virulent FVO parasite (in this animal model) to sustain until such a time that antibody levels fell below a certain threshold needed for effective neutralization. It is tempting to speculate that inducing similar antibody levels in humans could afford protection as parasite infection in humans is less virulent than seen in this *Aotus* model (Fig. 1a, Group 1). Interestingly, recent studies in this *Aotus* model showed that antibodies to AMA1 were boosted by malaria infection in animals vaccinated with AMA1,^22^ suggesting that natural infection may enhance vaccine responses. Our studies also raise caution against using solely GIA to predict in vivo outcomes (animals T3042, T3166, T3171 and T3174 having very different in vivo outcomes despite similar GIA). This could likely be due to the inability of the GIA assay to completely replicate the complex in vivo dynamics of host-parasite interactions. Instead GIA could be considered as the first step along the long path towards vaccine development.

Polymorphisms in AMA1 strongly influence the ability of AMA1 vaccines to neutralize non-vaccine-type parasites.^14, 25^ Interestingly, many of the polymorphisms are located in the loops surrounding the RON2 binding site.^29, 30^ This indicates that these loops are a major target of protective antibodies and the polymorphisms in these loops could help parasite escape antibody attack. Indeed, mAbs targeting these loops inhibit RON2L binding to AMA1 and block merozoite invasion.^11, 31^ This is in agreement with the current data that both Groups 2 and 3 showed a significantly higher GIA against homologous than heterologous parasites. Our previous study suggested that the superior vaccine efficacy induced by the complex may, at least in part, be due to a shift in antibody responses to some of these loops, possibly by stabilizing the conformation of these loops.^21^ Surprisingly, the *Pf* FVO AMA1–RON2L complex also enhanced the immunogenicity of certain conserved epitopes as demonstrated by a significant increase in GIA against heterologous parasites (Fig. 5d). Interestingly, inhibition of heterologous parasites by complex-induced antibodies was much higher than inhibition of homologous parasites by vaccination with AMA1-alone. The ability of the AMA1–RON2L complex vaccine to not only enhance vaccine efficacy against the homologous parasite, but also induce higher levels of antibodies against certain conserved epitopes is encouraging.

Another important aspect to come out of this study is the strong correlation between levels of AMA1-RON2 blocking antibodies and in vivo vaccine efficacy. The complex-induced enhancement in antibody quality may be, at least in part, due to a switch in the proportion of antibodies targeting the loops surrounding the RON2 binding site. It is also possible that some of the functional antibodies in these animals may target the AMA1-RON2 complex itself. Future studies will need to delineate these important epitopes that are the target of protective antibodies.

Our study suggests that the qualitative shift in antibody response induced by the complex is largely directed against AMA1. Previous studies have ruled out a major contribution of antibodies against the RON2L peptide in protection.^21^ However, it is conceivable that antibodies directed against the complex that are not detected by the currently available assays or other cellular mechanisms of immunity may contribute to protection. Due to the high virulence observed in the *Pf* FVO strain in the *Aotus* model, the need for high antibody titers for protection and the lack of a validated, readily available human-use adjuvant, we considered it prudent to use Freund’s adjuvant in this study. The next important step will be to determine if the complex can protect against human infection when formulated with an adjuvant safe for use in humans. Although GIA levels cannot predict in vivo outcome, they can be used as a qualitative assay to monitor the enhancement in the proportion of neutralizing antibodies. Several subunit vaccine candidates are under various stages of development,^32^ and the ones that have been tested in human trials have not shown much promise. An exciting recent development is the demonstration of RH5, a blood stage antigen, as a potential vaccine candidate.^22^ Indeed, levels of RH5 antibodies in malaria immune individuals were shown to correlate with protection.^33^ It remains to be seen if an RH5 vaccine can boost natural immunity as antibody levels are very low even in adults despite repeated infections.^33^ The challenge therefore is to sustain the pipeline with promising vaccine candidates that by themselves or in combination can be more efficacious. As AMA1 is one of the more immunogenic malaria antigens, it is tempting to speculate that immune responses to such next generation AMA1-based vaccines may be boosted through natural infections and a multi-allele AMA1 in complex with the conserved RON2L can protect against all parasites.

## Methods

### Recombinant protein

*P. falciparum* FVO strain AMA1 allele sequence was used in the vaccine. Expression and purification of recombinant FVO AMA1 full-length ectodomain (residues 25-546) in *P. pastoris* is described elsewhere.^34^ The recombinant protein was confirmed and validated to be of good quality as determined by various analytical methods described previously.^34^ SDS-PAGE and western blotting with a conformation-specific mAb (4G2) were used to verify correct protein folding.

### Peptide synthesis

Peptides were synthesized at Lifetein LLC (New Jersey, USA). Quality control performed include mass spectrometry for mass accuracy and high performance liquid chromatography for purity. Peptides used were >95% pure as determined using these methods.

### Surface plasmon resonance (SPR)

SPR measurements were made with a BIAcore T100 instrument at 25 °C according to the manufacturer’s instructions. Sensor CM5, amine coupling reagents, and buffers were purchased from GE Healthcare, Piscataway, NJ. The CM5 sensor chip was activated with N-hydroxysuccinimide and 3-(dimethylamino) propyl carbodiimide (EDC) for 7 min. Then, 40 µg mL^−1^ recombinant FVO AMA1-10 mM sodium acetate (pH 5.0) was injected for 7 min followed by blocking with 1 M ethanolamine (pH 8.5) for 7 min. A flow rate of 30 µL min^−1^ was used for all steps. For the binding assay, RON2L peptide was dissolved at different concentrations in 10 mM HEPES (pH 7.5)–0.5 mM EDTA–1 mM MgCl2–0.2% Tween 20. Binding at each concentration was done with an exposure of 2 min followed by 10 min for the dissociation phase. Regeneration was done with a 30-s pulse of 10 mM glycine HCl (pH 2.5). The kinetic data for RON2L binding were fitted to a two-step binding kinetic model. BIAcore T100 evaluation software was used for kinetic analysis.

### Animals

*Aotus* monkeys used in the study were housed-in female pairs and veterinary care was provided by NAMRU-6 attending veterinarian (MAJ Luis Lugo-Roman) and were monitored twice daily by animal caretakers. The monkeys were fed twice a day (morning and afternoon) with Iquitos Primate Center biscuits plus fresh fruits (bananas, apples). Room temperature (RT) was maintained at 24–27 °C, relative humidity between 30–70%, and a minimum of 10–15 air changes per hour and a light cycle consisting of a 12-h illumination followed by 12 h of dim red illumination. Nest boxes and perches (PVC tube pipes) were placed in the cages so the monkeys may “scent mark” and sit comfortably above the floor of the cage. Cleaning of the nest boxes and the cages were alternated on a weekly basis to maintain a “scent-marked” area in their cages at all times and additional toys were placed in the cages on a rotating basis for enrichment.

### Vaccines, animals, vaccinations and sample collection

AMA1–RON2L complex was prepared by mixing AMA1 and RON2L peptide in a 1: 3 gram ratio in PBS (pH 7.4). The mixture was incubated in the dark at RT for 30 min. All vaccinations were performed at the US Naval Medical Research Unit No. 6 (NAMRU-6), a facility accredited by the Association for Assessment and Accreditation of Laboratory Animal Care. A total of 22 malaria naïve, captive-bred, adult owl monkeys (*Aotus nancymaae*) were obtained from the Center for the Breeding and Conservation of Primates of Iquitos. They were randomized by sex and pretrial weight into three groups, Group1: PBS control (*n* = 6), Group 2: AMA1 alone (*n* = 8) and Group 3: AMA1–RON2L complex (*n* = 8). PBS, AMA1 alone or AMA1–RON2L complex were emulsified 1:1 in Freund’s complete (first immunization) or incomplete adjuvant (second and third immunizations) using two 5 mL syringes connected by double female luer lock (Smiths Medical# MX494) to form a thick emulsion. Three doses of the vaccine each containing 40 µg of AMA1 (Group 2) and 40 µg AMA1 + 120 µg RON2L (Group 3) in 0.5 mL were given subcutaneously (into the interscapular area) on study days 0, 21 and 42. Vaccination sites were monitored for adverse local reaction, and the hematocrit and weight of the animals was monitored at 3-week intervals. Plasma was collected under ketamine anesthesia from 2 mL EDTA-anticoagulated blood, 3 weeks after every vaccination. The study protocol was approved by NAMRU-6’s Institutional Animal Care and Use Committee (protocol number: NAMRU-6 14-01/NRD891), the Department of the Navy Bureau of Medicine and Surgery (NRD-748) and the Institut Nacional de Recursos Naturales (INRENA) at the Peruvian Ministry of Agriculture. (Resolucion Directoral No. 067-2014-SERFOR-DGGSPFFS). The experiments reported herein were conducted in compliance with the Animal Welfare Act and in accordance with the principles set forth in the “Guide for the Care and Use of Laboratory Animals”, Institute of Laboratory Animal Resources, National Research Council, National Academy Press, 2011.

### Parasite challenge

Four weeks after the final vaccination (study day 70), animals were challenged intravenously with 10^4^ FVO-strain *P. falciparum*-infected RBC collected freshly from a donor monkey. Parasitemia was measured by daily thin-film blood smears and hematocrit measurements were conducted on alternate days. Animals were treated with mefloquine (Roche Laboratories) when (i) parasite density reached ≥200,000 μL^−1^, (ii) when Hct fell to ≤25%, (iii) if SP upon reaching 40 days after challenge (study day 110). Animals that self-cured were monitored for 3 days for continued absence of parasite and were treated with mefloquine. Based on this criteria all 6 animals in Group 1 were mefloquine-treated for high parasitema. In Group 2, animal T3169 was treated for high parasitemia while T3042 and T3121 were treated due to anemia. T3095, T3118, T3171 and T3173 were treated 3 days after self-curing parasitemia. In Group 3, T3123 was treated due to anemia while T3160 died during self-curing parasitemia possibly due to anemia. Such occasional deaths have been recorded in this *Aotus* model of human malaria.^22, 35, 36^ T3174 developed anemia 2 days after self-curing and was also treated with mefloquine.

### ELISA

The assay was performed as described elsewhere.^37^ Briefly, ELISA plates were coated overnight with 1 µg mL^−1^ recombinant AMA1. *Pf*AMA1 allele-specific ELISA units were determined by first generating a standard curve using serially diluted IgG mixture containing anti-AMA1 antibodies. The reciprocal of the dilution giving an OD_405_ = 1 was used to assign ELISA units to standards and all samples were tested against the same standard as described.^37^

### Competition ELISA

The levels of AMA1-RON2 blocking antibodies were determined as follows. ELISA plates were coated overnight at 4 °C with 1 μg mL^−1^ RON2L peptide and blocked with 0.5% bovine serum albumin, 0.1% tween-20 in PBS (blocking buffer). Serial dilutions of plasma or purified IgG containing known antibody titers (AMA1 ELISA units, see above) prepared in blocking buffer were mixed with 2.5 μg mL^−1^ of recombinant FVO AMA1 and incubated for 30 min at RT. This mixture was applied to RON2L coated plates for 1 h at RT. After washing the plates, rabbit polyclonal AMA1 IgG was added to the plates at a 5 μg mL^−1^. The levels of bound AMA1 were measured using alkaline phosphatase-conjugated anti-rabbit secondary antibody at 1:3000 dilution. OD_405_ was measured and IC_50_ (antibody level, which inhibits 50% of AMA1 binding to RON2) for each sample was calculated using Graphpad Prism 5 software. Spearman correlation (*r*_s_) between AMA1-RON2 blocking antibodies and GIA (for IgG) and time to patency (for plasma) was analyzed by plotting of the IC_50_ (in Log10[EU] scale) using Graphpad Prism 5 software. Levels of blocking antibodies in the IgG (*n* = 8 per group) was tested once and the levels of blocking antibodies in the plasma samples (*n* = 7 per group) was tested in four independent experiments and the mean ± SEM of all experiments are shown.

### IgG antibody avidity ELISA

IgG antibody avidity was assessed by measuring the urea displacement method.^38^ ELISA was performed as described above. Individual IgG from animals in Group 2 (*n* = 5) and Group 3 (*n* = 8) were analyzed in duplicate. Following incubation and washing of IgG, an ascending concentration of urea (0 to 5 M) was added in duplicate wells. Plates were incubated for 15 min at RT and the concentration of urea needed to cause 50% reduction of the OD405 compared to the urea-free wells for each sample (i.e., the concentration of urea required to reduce the OD405 to 50% of that without urea = IC_50_) was used as a measure of avidity.

### *P. falciparum* parasite culture

All parasites strains were grown in RPMI 1640 supplemented with 25 mM HEPES and 50 μg mL^−1^ hypoxanthine (KD Medical), 0.5% Albumax (Invitrogen), 0.23% sodium bicarbonate (Gibco) using O + RBCs (Interstate Blood Bank, Jackson, TN) and monitored daily by Giemsa-stained blood smears as described.^39^

### Growth inhibition assay (GIA)

All assays for GIA were performed at the GIA reference center, NIAID, NIH. Plasma was heat inactivated for 20 min at 56 °C and pre-adsorbed against uninfected RBC. IgG was purified from plasma using Protein G sepharose (GE life sciences) using the low pH elution method and were neutralized immediately. Eluted IgG were dialyzed against RPMI 1640 (KD Medical) and concentrated to 10 mg mL^−1^. For performing GIA, IgGs at the desired concentrations were incubated with infected RBCs (0.3% parasitemia at 1% hematocrit) in a final volume of 40 µL for 40 h at 37 °C. A biochemical measurement using a *Pf* lactate dehydrogenase assay, as described previously, was used to quantify parasitemia.^23^

### Competition GIA

The GIA assay was performed as described above with some minor modifications. The ability of the various recombinant proteins (FVO AMA1, 3D7 AMA or reduced and alkylated FVO AMA1) to adsorb the inhibitory activity of IgG was measured by pre-incubating the IgG with saturating concentration (2 µM) of recombinant proteins for 30 min at RT as determined previously.^21^

### Statistical analysis

Primary end point for vaccine efficacy was analyzed by Mantel-Cox test of time-to-patency of animals in Group 2 and Group 3. Secondary efficacy outcome was measured by comparing log cumulative parasitemia between Groups 2 and 3 by Mann–Whitney test. Comparison of the levels of anti-AMA1 antibodies in the plasma, purified IgG and comparison of GIA between the two groups were also performed by Mann-Whitney test. Association between immunological parameters, in vitro and in vivo outcome202s were assumed to be non-Gaussian distribution and were analyzed by Spearman’s rank correlation. To determine whether there was a quality difference between Group 2 and 3 IgGs in GIA, a multiple regression analysis was performed. GIA level was used as a response variable, and AMA1 antibody level and Group as explanatory variables. For the relationship between levels AMA1–RON2L blocking antibodies and in vitro GIA or in vivo time-to-patency, IC_50_ was calculated using a non-linear fit of normalized dose response curves for each sample containing a known amount (ELISA unit) of anti-AMA1 antibodies and analyzed by Spearman’s rank correlation.

The attending veterinarian and technicians who assisted or performed vaccination, parasite challenge, parasitemia counts, GIA, ELISA were all blinded to the vaccination groups. Data were unblinded during analysis by the primary investigators.

### Data availability

All relevant data are available from the authors

### Disclaimer

The views expressed in this article are those of the authors and do not necessarily reflect the views of USAID nor the official policy or position of the Department of the Navy, Department of Defense or the US Government.

### Copyright statement

Several authors are employees of the U.S. Government and this work was prepared as part of official duties. Title 17 U.S.C. §105 provides that ‘Copyright protection under this title is not available for any work of the United States Government.’ Title 17 U.S.C. §101 defines a U.S. Government work as a work prepared by an employee of the U.S. Government as part of that person’s official duties.

## Electronic supplementary material

Supplementary Figure 1

Supplementary Figure 2

Supplementary Figure 3

Supplementary Figure 4

Supplementary Figure Legends
