# Supplementary figures and images for: A malaria vaccine protects Aotus monkeys against virulent Plasmodium falciparum infection
Source: NPJ Vaccines. 2017 May 22;2:14. doi: 10.1038/s41541-017-0015-7 (PMC5551459; doi:10.1038/s41541-017-0015-7)

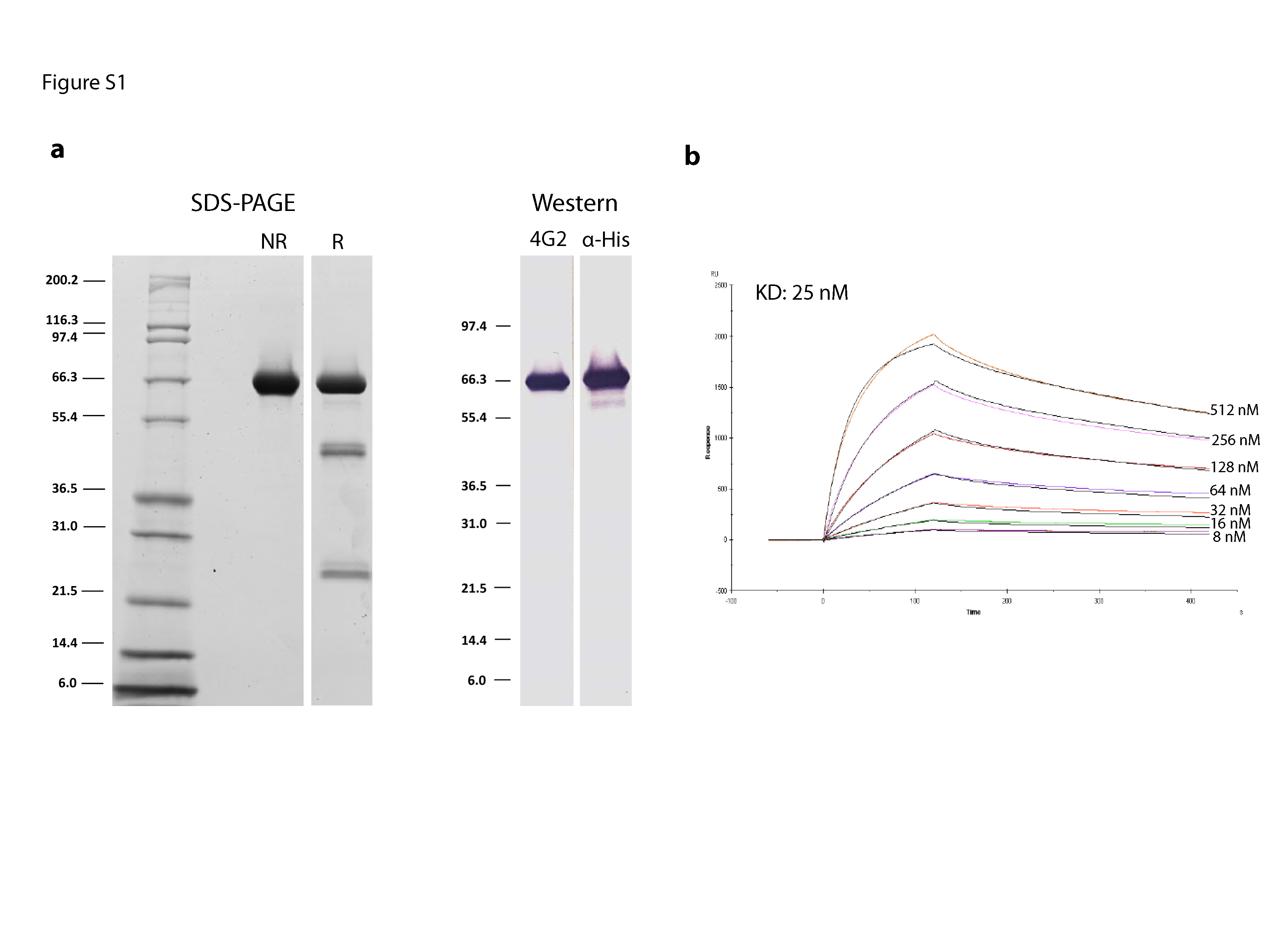

Supplement: Supplementary file 1 — Supplementary Figure 1 [file 41541_2017_15_MOESM1_ESM.tif]

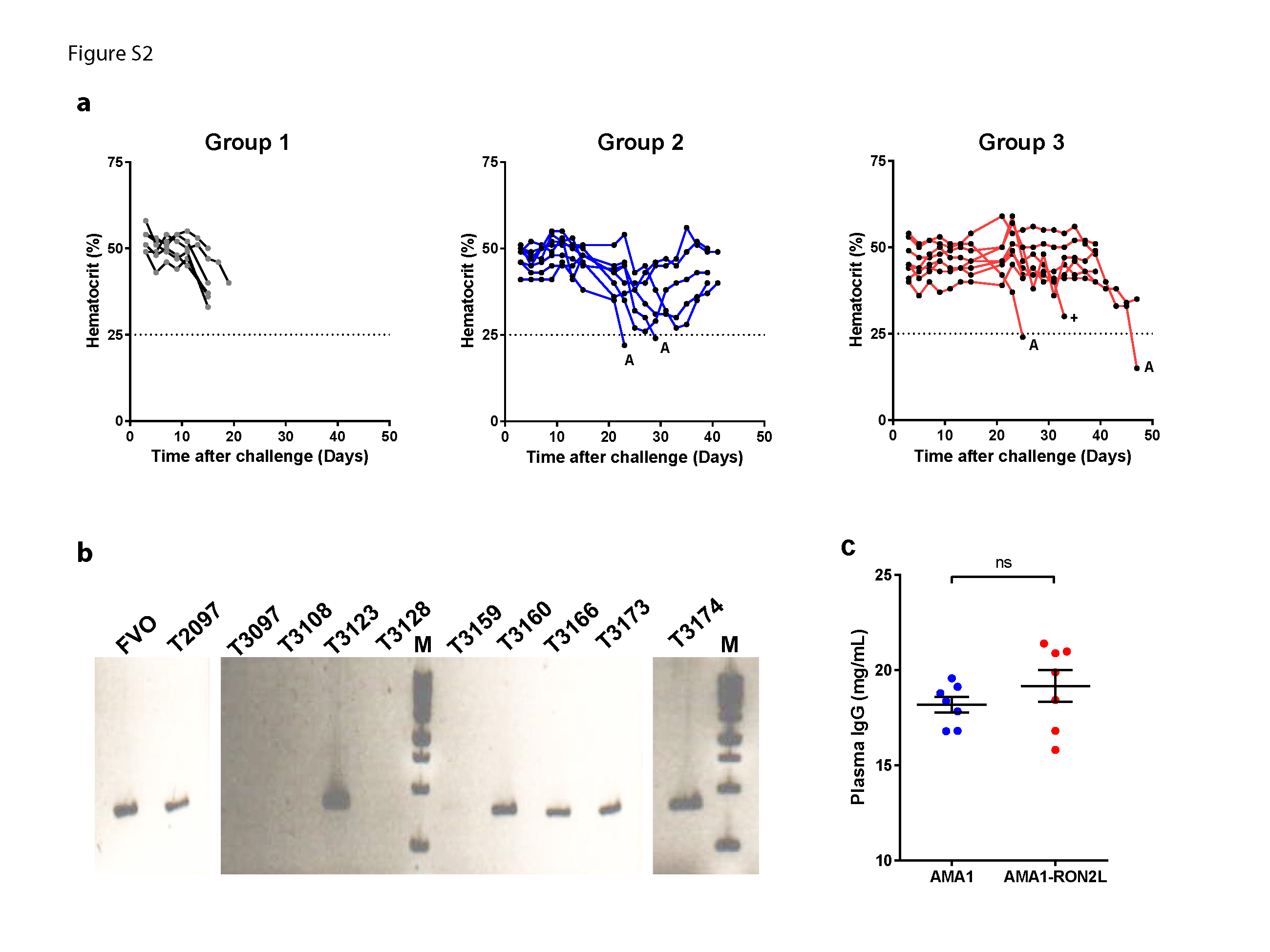

Supplement: Supplementary file 2 — Supplementary Figure 2 [file 41541_2017_15_MOESM2_ESM.tif]

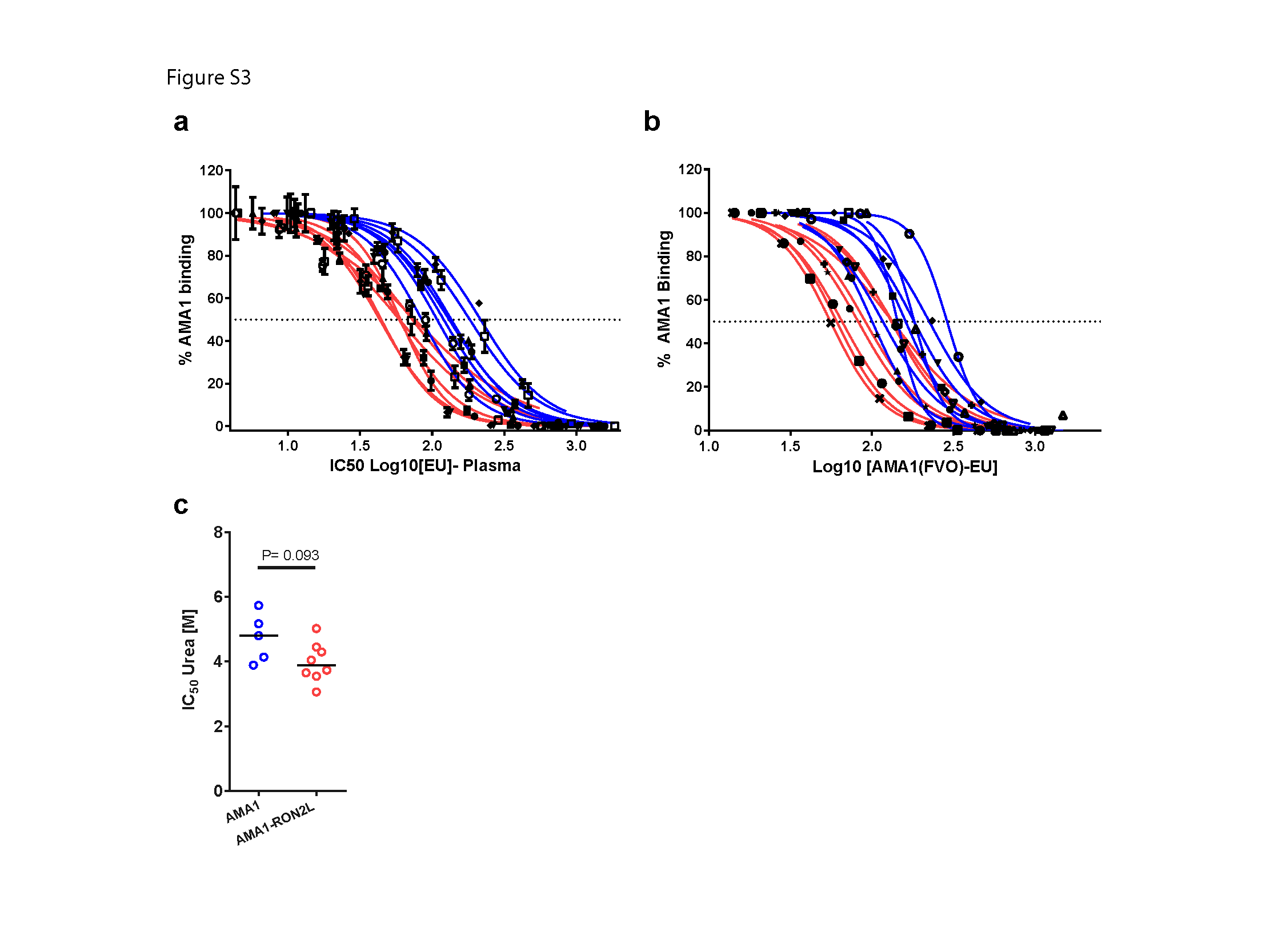

Supplement: Supplementary file 3 — Supplementary Figure 3 [file 41541_2017_15_MOESM3_ESM.tif]

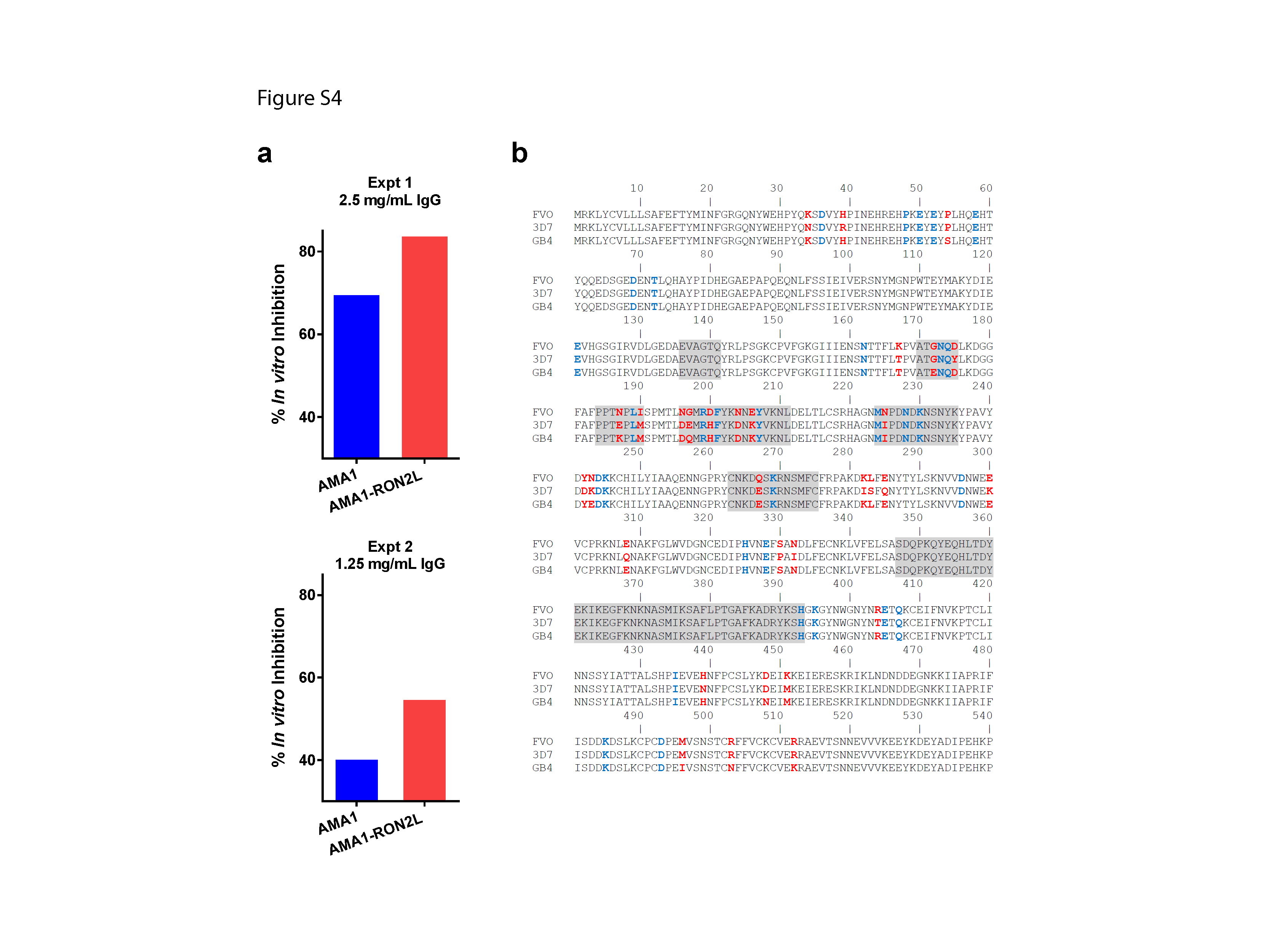

Supplement: Supplementary file 4 — Supplementary Figure 4 [file 41541_2017_15_MOESM4_ESM.tif]
