# Supplementary material for: A malaria vaccine protects Aotus monkeys against virulent Plasmodium falciparum infection
Source: NPJ Vaccines. 2017 May 22;2:14. doi: 10.1038/s41541-017-0015-7 (PMC5551459; doi:10.1038/s41541-017-0015-7)
Supplement: Supplementary file 5 — Supplementary Figure Legends [file 41541_2017_15_MOESM5_ESM.docx]

**Supplementary Figure Legends**

**Figure S1**. **Quality of recombinant FVO AMA1 used in the vaccine.** **(a)** SDS-PAGE (left) of recombinant FVO AMA1 under non-reducing (NR) and reducing (R) conditions. Western blot (right) under non-reducing conditions were performed using conformation-specific AMA1 mAb 4G2 to and anti-His antibody to detect the His tag at the c-terminus of the recombinant protein. **(b)** Surface plasmon resonance demonstrating AMA1-RON2L complex formation. Various concentrations (nM) of RON2L peptide used to determine the K_D_ are indicated. The curves were fitted using the two-state binding model.

**Figure S2.** Assessment of hematocrit **(a)** and PCR detection of blood stage parasites in selected animals before treatment **(b)**. Hematocrit was followed every other day after challenge until the animals were treated for high parasitemia or when the hematocrit dropped below 24%. A; animals treated due to anemia, +; animal that died possibly due to declining hematocrit. PCR was performed from genomic DNA prepared from 50uL of packed RBCs to detect possible sub-patent parasitemia in the four animals from Group 3 (T3097, T3108, T3128 and T3159) that remained thin smear negative on day 40. Genomic DNA prepared from FVO parasites grown in culture was used as positive control. DNA from blood collected on day 8 (T2097), day 21 (T3123 and T3173), day 28 (T3160), day 36 (T3166 and T3174) after parasite challenge at different levels of parasitemia (290 – 74,000 parasites/ µL blood) was used as positive controls. **(c)** Total IgG concentration from plasma of animals immunized with AMA1 alone (Group 2) and AMA1-RON2L complex (Group 3) were compared by Mann-Whitney test (P = 0.256). Data are shown for individual animals and represented as mean ± SEM.

**Figure S3.** **Correlates of protection.** Competition assay to measure level of AMA1-RON2L blocking antibodies in plasma **(a)**, purified IgG **(b)**. Serial 2-fold dilutions of plasma (mean ± sem of three independent experiments, n=7 animals each form Group 2 and Group 3) or IgG (n= 8 animals each from Group 2 and Group 3 from one experiment) was mixed with recombinant FVO AMA1 and used to measure the level of AMA1-RON2L blocking antibodies by ELISA. **(c)** Determination of relative avidity of the purified IgG from animals in Group 2 (n=5) and Group 3 (n=8).

**Figure S4**. ***In vitro* growth inhibition assay against heterologous GB4 parasites.** **(a)** GIA was against heterologous GB4 parasites was measured using pooled IgG from Group 2 and Group 3 in two independent experiments performed using 2.5 mg/mL and 1.25 mg/mL total IgG respectively. The mean of each of the two experiments performed in duplicate is shown. **(b)** Sequence comparison of domains 1, 2 and 3 of AMA1 from FVO, 3D7 and GB4, the three parasites used in GIA assays in this study is shown. Polymorphic residues in AMA1 that are conserved between the three parasites are shown in blue and the residues that differ are shown in red. Shaded regions indicate the loops in domain 1 and domain 2 of AMA1 that surround the RON2L binding.
